# Validation of the Chinese version of the PHQ-15 in a tertiary hospital

**DOI:** 10.1186/s12888-016-0798-5

**Published:** 2016-04-05

**Authors:** Lan Zhang, Kurt Fritzsche, Yang Liu, Jian Wang, Mingjin Huang, Yu Wang, Liang Chen, Shanxia Luo, Jianying Yu, Zaiquan Dong, Liling Mo, Rainer Leonhart

**Affiliations:** Mental Health Center, West China Hospital of Sichuan University, No. 28 Dianxin S. St., Chengdu, Sichuan 610041 P. R. China; Department of Psychosomatic Medicine and Psychotherapy, University Medical Centre Freiburg, Hauptstr. 8, D-79104 Freiburg, Germany; Institute of Psychology, University of Freiburg, Freiburg, Germany

**Keywords:** Patient Health Questionnaire (PHQ-15), Somatic symptoms, Item response theory analysis, Validation, China

## Abstract

**Background:**

This study aimed to investigate the reliability and validity of the Chinese version of the Patient Health Questionnaire (PHQ-15) in a tertiary hospital.

**Methods:**

Using a cross-sectional study design, the Chinese version of the PHQ-15 was administered to a total of 1329 inpatients. To examine the discriminant validity of this questionnaire, we investigated the correlation of the PHQ-15 score with sociodemographic data and the PHQ-9 and GAD-7 scale scores. Exploratory factor analysis was performed to assess the internal consistency of the PHQ-15. To evaluate the consistency of this questionnaire with item response theory (IRT), IRT analysis was performed.

**Results:**

The Chinese version of the PHQ-15 showed good reliability (Cronbach’s alpha = 0.83). The correlations of the PHQ-15 scores with the PHQ-9 depression scale scores (*r* = 0.565) and the GAD-7 anxiety scale scores (*r* = 0.512) were moderate; these results suggested that the PHQ-15 had discriminant validity. We identified three factors, referred to as “cardiopulmonary,” “gastrointestinal,” and “pain/neurological,” which explained 56 % of the total variance. A second-order factor analysis including these three factors produced an acceptable model. Several items (4, 8 and 11) displayed extreme floor effects. Additionally, item 4 displayed a very small variance of 0.35 and showed very small differences in its thresholds based on IRT analysis.

**Conclusions:**

The PHQ-15 scale had good reliability and high validity to detect patients with high somatic symptom severity in a Chinese tertiary hospital. Several of the current findings were consistent with previous research on the PHQ-15 in Western countries and in China. To improve the diagnostic quality of this questionnaire, items 4, 8 and 11 can be omitted.

**Electronic supplementary material:**

The online version of this article (doi:10.1186/s12888-016-0798-5) contains supplementary material, which is available to authorized users.

## Background

Patients with multiple distressing somatic symptoms are prevalent in primary, secondary and tertiary care settings [1–4]. The Patient Health Questionnaire (PHQ)-15 [5] is an economical, self-administered instrument that has been used as a screening tool in several studies. Moreover, the PHQ-15 has been suggested by the DSM-5 Workgroup on Somatic Symptom Disorders (SSD) to serve as a measure of somatic symptom severity for the classification of SSD [6, 7].

The PHQ-15 has been developed from its precursors, the “Primary Care Evaluation of Mental Disorders” (PRIME-MD) [8] and the “PRIME-MD Patient Health Questionnaire” (PRIME-MD PHQ) [9]. Previous studies [5, 10–12] suggest that individual somatic symptoms frequently cluster into 4 groups: cardiopulmonary, gastrointestinal, pain, and general.

Among 40 self-reported somatic symptom scales investigated in a review, the PHQ-15 and the 12-item Symptom Checklist–90 somatization scale [13] were identified as the most appropriate measures for large-scale studies because of their well-established psychometric properties, relevance to symptoms, brevity, and availability in multiple languages [11].

### The PHQ-15 in Hong Kong and Mainland China

Historically, there has been a popular belief that Asians manifest a lower prevalence of mood and anxiety disorders than their Western counterparts because they are more prone to experiencing and manifesting distress via somatic pathways [14–16]. Among Chinese patients receiving psychiatric services, somatic symptoms such as pain, insomnia and fatigue have been associated with depressive and anxiety disorders [17].

The validity and reliability of the Chinese version of the PHQ-15 [18] were examined in the general population of Hong Kong. The Hong Kong version of the PHQ-15 exhibited satisfactory internal consistency (Cronbach’s alpha = 0.79) and stable 1-month test-retest reliability. Somatic symptom severity positively associated with functional impairment and health service use.

In mainland China, the validity and reliability of the PHQ-15 were tested in the outpatient clinics of general hospitals in Shanghai [19]. Cronbach’s alpha was 0.73, and the test-retest reliability coefficient was 0.75. There were moderate positive correlations between the PHQ-15 score and anxiety and depression values.

No PHQ-15 data are available for tertiary hospital inpatients in China, and no item response theory (IRT) analyses have been performed. IRT is a probabilistic test theory that represents a strong paradigm for the analysis of tests or questionnaires. Compared to the “simpler” classical test theory, IRT does not assume that each item is equally difficult.

Furthermore, we found several inconsistencies in translation among the English, Hong Kong and Shanghai versions of the PHQ-15 (see Methods section).

The objective of the present study was to assess the validity of the Chinese version of the PHQ-15 for the detection of distressing somatic symptoms in a sample of inpatients at a tertiary hospital.

We aimed to answer the following research questions:

1. What somatic symptoms are reported most often by patients?
2. What is the internal consistency and discriminant validity of the Chinese PHQ-15?
3. Is the PHQ-15 consistent with IRT?

## Methods

### Study design

We conducted an observational cross-sectional survey. The study was initiated under normal clinical conditions on a random day in October 2013.

Participants in this study were inpatients recruited from 10 departments (oncology, cardiology, respiratory medicine, rehabilitation, geriatrics and gerontology, general practice, pain management, thyroid and breast surgery, rheumatology, and hepatic surgery) of the West China Hospital of Sichuan University. The West China Hospital of Sichuan University is a “3 A hospital,” indicating that it meets the highest standards in China. The West China Hospital provides primary, secondary and tertiary care and has a full complement of services, including the departments mentioned above.

All inpatients of these departments were considered to be potential participants in our study. The following inclusion criteria were used: (1) treatment as an inpatient in the selected wards; (2) sufficient language skills to understand the questionnaires; and (3) informed consent to participate in the research. Exclusion criteria were (1) discharge from the hospital on the day of survey completion and (2) inability to independently complete the self-reported questionnaire due to serious physical debilitation or mental status. The investigators were well trained medical doctors, nurses or medical students. A pilot study was performed in advance to confirm the feasibility of the study, e.g., that the patients would agree to participate and would understand the questionnaires. The investigators collected the questionnaires from the patients.

The validation of the PHQ-15 is a component of a larger project investigating the prevalence and recognition of inpatients with emotional distress and their treatment needs at a general hospital.

### Assessment instruments

#### PHQ-15

The PHQ-15 is a self-administered somatic symptoms subscale derived from the full PHQ [5.9]. The PHQ-15 includes 15 prevalent somatic symptoms or symptom clusters that represent over 90 % of the symptoms observed in primary care (exclusive of self-limited upper respiratory symptoms such as cough, nasal symptoms, sore throat, and ear ache) [5]. The patients were asked to rate the severity of their symptoms during the previous 4 weeks on a 3-point scale as either 0 (“not bothered at all”), 1 (“bothered a little”) or 2 (“bothered a lot”). Two items consisted of questions regarding “feeling tired and having little energy” and “trouble sleeping”; these items are in the depression module of the PHQ-15.

The classification of somatic symptom severity included minimal (0–4), mild (5–9), moderate (10–14) and severe (15–30). The total symptom severity score ranged from 0 to 30.

The reliability of the PHQ-15 was initially supported by the results of one study of 6000 patients from general internal medicine and family practice clinics in Phoenix [5]. In that study, the PHQ-15 demonstrated good internal consistency (Cronbach’s alpha = 0.80) and was related to criterion indices or physical dysfunction, self-reported disability days, clinic visits, and the amount of difficulty that the patients attributed to their symptoms. Furthermore, linear regressions were performed to examine the ability of the PHQ-15, along with other variables, such as depression scores and medical comorbidities, to independently predict clinical outcomes (e.g., bodily pain and physical functioning).

To determine prevalence rates, a cut-off score of ≥ 10 was used because the range of 10 to 30 reflects moderate to high somatic symptom severity. The selection of this cut-off score was based on previous studies [20, 21].

Each item is measured using a ranking scale; therefore, one open question is whether the sum of these data can be interpreted as metric data. Additionally, the merits of a 3-point scale compared with a 2-point scale are discussed.

The PHQ-15 has been translated into other languages and has been examined in samples from many countries, e.g., Saudi Arabia, Germany, Spain, Belgium, Korea, and the Netherlands. This evidence offers the potential for comparisons between ethnic groups.

#### Translation of the PHQ-15

This study is part of the Sino-German research cooperation, which was started in 2010. Workshops and a multicenter study on illness perception and illness attribution in patients with somatoform disorders were funded by a grant from the Sino-German Center of Research Promotion in Beijing. A working group of three native Chinese speakers who resided in Germany and were fluent in written and spoken English and German (one psychiatrist, one psychologist, and one educator) was established to revise the Chinese version of the PHQ-15. One translator regularly participated in project meetings. Translations were discussed during the project meetings [22, 23].

We used the Chinese version translated from English to Mandarin by colleagues from Shanghai Mental Center [19]. Because “stomach pain” (胃痛) in item 1 was narrowly translated to mean “gastric pain” in the mainland Chinese version, we changed this to “stomach and abdominal pain” (胃痛或肚痛), in accordance with the suggestions of Lee et al. [18]. In item 8, “fainting spells” was translated to “occasional fainting” (偶尔昏晕过去) in the Shanghai version, but we preferred the Hong Kong wording of “brief fainting” [短時間暈倒 (Cantonese), 短时间晕倒 (Mandarin)]. Please see Additional file 1.

Other than these slight changes, back-translation of the Chinese PHQ-15 showed perfect concordance with the English language version of the PHQ-15.

The Mandarin version of the PHQ-15 used in this study can be provided upon request to the corresponding author.

### Depression scale (PHQ-9)

The Patient Health Questionnaire-9 (PHQ-9) assesses each of the nine DSM-IV depression criteria on a scale of “0” (not at all) to “3” (nearly every day) [24]. The PHQ-9 demonstrated acceptable psychometric properties for the screening of patients with late-life depression in Chinese primary care settings, as this questionnaire showed a sensitivity of 0.86 and a specificity of 0.77 [25].

### General Anxiety Disorder (GAD-7)

A seven-item anxiety scale (GAD-7) was used to assess the severity of generalized anxiety [26]. In a Chinese general hospital population, this instrument showed good reliability and good criterion, construct, factorial, and procedural validity [27].

### Statistical analyses

Using IBM SPSS (23.0), STATA 14 and MPlus 7.3 software, a single sample was analyzed. For descriptive analyses of the quantitative variables, mean, standard deviation and range were calculated, and for analyses of the qualitative variables, frequencies and percentages were used. The distribution of the total scores obtained using Chinese version of the PHQ-15 was studied, and the percentage of patients with each of the possible total scores was calculated.

Three types of analyses were performed to evaluate validity. First, to examine the discriminant validity of the PHQ-15, we investigated the correlations of the PHQ-15 scores with sociodemographic data and the PHQ-9 and GAD-7 scale scores. Based on the results from previous studies of the PHQ-15, we expected that women would have higher somatic symptom severity (SSS) scores than men and that the SSS scores would increase with increasing age and decreasing education level [19]. Second, reliability was analyzed in terms of internal consistency using Cronbach’s alpha coefficient for the total scale score. Exploratory factor analysis was performed to reveal the structure of the internal consistency of the PHQ-15. Finally, IRT analysis was performed to assess the thresholds of the items because each item had only three answer options and could only be interpreted as rank data.

Statistical analyses were conducted using an alpha level of 1 % to avoid alpha inflation resulting from multiple tests.

## Results

### Description of the sample

Of the 1662 inpatients approached in the 10 departments, 151 patients were excluded based on the exclusion criteria, and 149 patients refused to participate in the study. The main reasons that patients gave for their non-participation were lack of time (*n* = 27) or interest (*n* = 60). The final sample consisted of 1362 subjects, corresponding to an overall response rate of 90.1 %. Patients for whom more than 15 % of the data were missing were excluded. Therefore, 1329 eligible patients were included in our study.

The mean total score on the PHQ-15 was 6.79, with a standard deviation of 4.94 (minimum = 0; maximum = 28). We divided the PHQ-15 data from the sample into two groups: the somatoform symptom (SOM) - group (PHQ-15 score <10, *n* = 960, mean = 4.34, SD = 2.80) and the SOM+ group (PHQ-15 score **≥** 10, *n* = 369, mean = 13.18, SD = 3.28).

The PHQ-15 score moderately correlated with the PHQ-9 score (*r* = 0.565) and the GAD-7 score (*r* = 0.512). The Spearman rank correlation coefficients of the PHQ-15 score with income (*r* = −0.069) and education levels (*r* = −0.075) were near zero.

The sociodemographic data of the sample are presented in Table 1. Based on an alpha level of 0.1, there were no significant differences in sociodemographic characteristics between the SOM- and SOM+ groups. The distributions of education level and income were comparable to the distributions observed in other studies performed at the general hospitals in China [28–30].

**Table 1:** Sociodemographic characteristics

Variable	PHQ-15 score (*n* = 1329)	PHQ-15 score <10 (*n* = 960)	PHQ-15 score ≥10 (*n* = 369)	*t/Chi* ^*2*^ *(df) p*
Age				
Mean (SD)	53.76 (16.21)	53.56 (16.02)	54.26 (16.25)	−0.700 (1320) 0.484
Gender				
Male	58.1 %	60.0 %	53.0 %	5.370 (1) 0.020
Female	41.9 %	40.0 %	47.0 %	
Marital status				
Unmarried	9.1 %	9.6 %	7.9 %	2.233 (1) 0.525
Married	87.8 %	87.8 %	88.3 %	
Divorced	1.1 %	0.9 %	1.6 %	
Widowed	1.9 %	1.8 %	2.2 %	
Race				
Han	95.5 %	4.2 %	5.2 %	0.603 (1) 0.437
Others	4.5 %	95.8 %	94.8 %	
Education level				
Elementary school	2.1 %	2.0 %	2.5 %	14.958 (5) 0.011
Junior high school	16.4 %	14.3 %	21.9 %	
High school or secondary technical school	26.6 %	26.3 %	27.7 %	
College	23.8 %	24.9 %	20.8 %	
Undergraduate	17.5 %	17.9 %	16.4 %	
Graduate and above	13.6 %	14.6 %	10.7 %	
Income				
5000 yuan and below	14.1 %	12.8 %	17.5 %	14.717 (5) 0.012
5000–9999 yuan	13.2 %	12.0 %	16.1 %	
10,000–29,999 yuan	16.2 %	15.5 %	18.0 %	
30,000–49,999 yuan	21.1 %	22.0 %	18.9 %	
50,000–99,999 yuan	20.8 %	22.6 %	16.3 %	
100,000 yuan and above	14.6 %	15.1 %	13.2 %	

All percentages correspond to the n shown at the top of the given column

The clinical data are presented in Table 2.

**Table 2:** Clinical measures

	*N* = 1329	SOM- group (PHQ-15 score <10) *N* = 960	SOM+ group (PHQ-15 score ≥10) *N* = 369	
Categorical variables		n (%)	n (%)	*Chi* ^*2*^ *(df)/p*
Smoking				
Never	755 (57.5 %)	534 (56.3 %)	221 (60.5 %)	4.234 (2) 0.120
Once, has quit smoking	465 (35.4 %)	339 (35.8 %)	126 (34.5 %)	
Still smoking	93 (7.1 %)	75 (7.9 %)	18 (4.9 %)	
Use of alcohol				
Never drink	709 (54.3 %)	502 (53.2 %)	207 (57.3 %)	4.352 (3) 0.226
Social drinking	358 (27.4 %)	274 (29.0 %)	84 (23.3 %)	
Prior daily drinking (now stopped)	211 (16.2 %)	149 (15.8 %)	62 (17.2 %)	
Drink almost every day (more than 3 days/week)	27 (2.1 %)	19 (2.0 %)	8 (2.2 %)	
Department				
Respiratory Medicine	142 (10.7 %)	97 (10.1 %)	45 (12.2 %)	19.083 (9) 0.024
Internal Cardiology	153 (11.5 %)	107 (11.1 %)	46 (12.5 %)	
Rheumatology	75 (5.6 %)	42 (4.4 %)	33 (8.9 %)	
Pain Management	30 (2.3 %)	24 (2.5 %)	6 (1.6 %)	
Rehabilitation	119 (9.0 %)	84 (8.8 %)	35 (9.5 %)	
Oncology	527 (39.7 %)	399 (41.6 %)	128 (34.7 %)	
Geriatrics and Gerontology	115 (8.7 %)	79 (8.2 %)	36 (9.8 %)	
Thyroid and Breast Surgery	46 (3.5 %)	38 (4.0 %)	8 (2.2 %)	
Hepatic Surgery	61 (4.6 %)	44 (4.6 %)	17(4.6 %)	
General Practice	61 (4.6 %)	46 (4.8 %)	15 (4.1 %)	
Continuous variables MANOVA	M (SD)	M (SD)	M (SD)	*F (df1,df2) p* (partial Eta^2^)
PHQ-9 score	7.59 (5.05)	6.16 (4.38)	11.31 (4.79)	349.245 (1,1327) <0.001 (0.208)
GAD-7 score	5.12 (4.75)	3.86 (3.85)	8.42 (5.26)	301.935 (1,1327) <0.001(0.185)

All % are column percentages

A comparison of all departments showed no significant difference in the mean PHQ-15 score between the SOM- and SOM+ groups considering an alpha level of 1 % (F (9.1319) = 1.904, *p* = 0.048, partial Eta^2^ = 0.013). Significant differences (based on MANOVA) in the mean PHQ-9 and GAD-7 scale scores between the SOM- and SOM+ groups were found.

### Item and scale characteristics

The distributions of the items displayed extreme floor effects (see Table 3); in particular, item 4 showed a frequency of 91.1 % for the null, compared to 7.4 % for item one and 1.4 % for item two. Other items, such as items 8 and 11, displayed comparable floor effects. Item 4 showed a very small variance of 0.35. Other items displayed greater variances, but none of the items displayed an ideal difficulty of approximately 1 on a scale from 0 to 2. An ideal difficulty of 1 would be valuable for improving the reliability of the questionnaire because this is a requirement for variance and for a large Cronbach’s alpha.

**Table 3:** Descriptive statistics for the PHQ-15

Item	Mean score (SD)	Frequency of score 0 (%)	Frequency of score 1 (%)	Frequency of score 2 (%)	Part-hole corrected item-to-scale correlation
1	0.37 (0.59)	917 (69.0 %)	336 (25.3 %)	76 (5.7 %)	0.489
2	0.53 (0.64)	733 (55.2 %)	488 (36.7 %)	108 (8.1 %)	0.505
3	0.60 (0.70)	703 (52.9 %)	458 (34.5 %)	168 (12.6 %)	0.396
4	0.10 (0.35)	1211 (91.1 %)	99 (7.4 %)	19 (1.4 %)	0.146
5	0.39 (0.57)	869 (65.4 %9	400 (30.1 %)	60 (4.5 %)	0.488
6	0.41 (0.59)	850 (64.0 %)	409 (30.8 %)	70 (5.3 %)	0.501
7	0.48 (0.59)	758 (57.0 %)	509 (38.3 %)	62 (4.7 %)	0.508
8	0.20 (0.48)	1109 (83.4 %)	175 (13.2 %)	45 (3.4 %)	0.380
9	0.44 (0.59)	815 (61.3 %)	444 (33.4 %)	70 (5.3 %)	0.526
10	0.40 (0.62)	891 (67.0 %)	342(25.7 %)	96 (7.2 %)	0.487
11	0.21 (0.48)	1099 (82.7 %)	184 (13.8 %)	46 (3.5 %)	0.318
12	0.60 (0.67)	672 (50.6 %)	522 (39.3 %)	135 (10.2 %)	0.435
13	0.58 (0.65)	685 (51.5 %)	522 (39.3 %)	122 (9.2 %)	0.517
14	0.77 (0.66)	479 (36.0 %)	675 (50.8 %)	175 (13.2 %)	0.556
15	0.72 (0.72)	578 (43.5 %)	542 (40.8 %)	209 (15.7 %)	0.489

The PHQ-15 displayed a Cronbach’s alpha of 0.833. Excluding item 4 slightly increased Cronbach’s alpha to 0.837. The item-to-item correlations were in the range of 0.32 to 0.56. The item-to-item correlations with item 4 were less than 0.15, and the item-to-item correlations of 6 items exceeded 0.50.

The deduced determination coefficients showed a common variance with the PHQ-15 score of 31.9 % for the PHQ-9 score and 26.2 % for the GAD-7 scale score. The discriminant validity of the PHQ-15 is therefore acceptable because the PHQ-15 measures different constructs than the PHQ-9 or the GAD-7 scale in this sample. Cronbach’s alpha was 0.908 for the PHQ-9 and 0.815 for the GAD-7 scale.

### Factorial validity

For internal consistency, we performed exploratory factor analysis on the categorical data using MPlus software (see Table 4). All subjects were included in this analysis. By adopting the Kaiser criterion (Eigenvalue >1), three factors were extracted; these factors accounted for 55.97 % of the total variance. The Eigenvalues of the three factors were as follows: factor 1 = 6.026, factor 2 = 1.279 and factor 3 = 1.091. Based on this factor structure, the items loading the 3 factors may be termed “cardiopulmonary”, “gastrointestinal” and “pain”. Thirteen items of the PHQ-15 loaded on only one of the factors; in contrast, items 1 and 2 cross-loaded on two of the factors. The Chi-Square Test of Model Fit showed that the sample size was acceptable (Chi^2^ = 371.064, df = 63, *p* < 0.0001). The root mean square error of approximation (RMSEA) of 0.061 was acceptable (90 % C.I.: 0.055 - 0.067). The Comparative Fit Index (CFI) was adequate (0.961), and the Tucker-Lewis Index (TLI) was acceptable (0.935). The standardized root mean square residual (SRMR) was approximately 0.048. The geomin-rotated factors showed correlations between 0.418 and 0.531.

**Table 4:** Geomin-rated factor loadings

Item	Factor 1	Factor 2	Factor 3	Residual variances
1	0.406^a^	−0.058	0.435^a^	0.520
2	0.392^a^	0.043	0.331^a^	0.581
3	0.363^a^	−0.014	0.251^a^	0.726
4	0.359^a^	−0.044	−0.002	0.886
5	0.838^a^	−0.092	0.006	0.367
6	0.507^a^	0.201^a^	0.082	0.534
7	0.653^a^	0.017	0.106^a^	0.483
8	0.588^a^	0.218^a^	−0.130^a^	0.550
9	0.176^a^	0.724^a^	−0.001	0.309
10	0.005	0.853^a^	0.053	0.228
11	0.485^a^	0.089	−0.018	0.721
12	0.091	0.041	0.566^a^	0.597
13	0.179^a^	−0.001	0.646^a^	0.441
14	−0.021	0.272^a^	0.658^a^	0.362
15	0.079	0.245^a^	0.457^a^	0.576

^a^significant at the 5 % level

Ten out of the fifteen variables in this model had significant double- or, in some cases, triple-loadings. Some of these double-loadings had nearly the same values. However, a one-factor model displayed a worse fit (Chi^2^ = 928.208, df = 90, *p* < 0.0001). The RMSEA of 0.084 was not acceptable (90 % C.I.: 0.079 - 0.089). The CFI was marginal (0.894), and the TLI was marginal or unacceptable (0.876). The SRMR was approximately 0.077. Because the three factors moderately correlated, the one-factor-model displayed a poor fit, and because the authors of the questionnaire used the sum of all items as an outcome, we conducted second-order factor analysis considering these three factors and a second-order factor. The Chi-Square Test of Model Fit considered the large sample size to be acceptable (Chi^2^ = 451.988, df = 85, *p* < 0.0001. The RMSEA of 0.057 was acceptable (90 % C.I.: 0.052 - 0.062). The CFI was adequate (0.954), and the TLI was acceptable (0.943). The weighted root mean square residual (WRMR) was approximately 1.554. For this model, double-loading for items 1 and 2 was supposed. The R^2^ of the three factors was high (Factor 1 = 0.733, Factor 2 = 0.622, and Factor 3 = 0.684).

### IRT analysis

IRT analysis of the partial credit model showed that all of the items suited the model. The problematic item 4 displayed two thresholds in the appropriate order, but these thresholds did not markedly differ (2.498 vs 2.583). All of the other items showed greater differences in their thresholds and showed adequate results based on IRT analysis (see Table 5).

**Table 5:** Thresholds for the PHQ-15 based on IRT analysis

	Diff.	Coef.	Std. Err.	z	*P* > z	[95 % Conf.	Interval]
Item 1	1 vs 0	0.994	0.069	14.44	<0.001	0.859	1.129
	2 vs 1	2.132	0.124	17.13	<0.001	1.888	2.376
Item 2	1 vs 0	0.355	0.061	5.76	<0.001	0.235	0.476
	2 vs 1	2.024	0.108	18.75	<0.001	1.812	2.235
Item 3	1 vs 0	0.326	0.063	5.19	<0.001	0.203	0.448
	2 vs 1	1.532	0.091	16.83	<0.001	1.354	1.710
Item 4	1 vs 0	2.498	0.114	21.89	<0.001	2.274	2.721
	2 vs 1	2.583	0.228	11.34	<0.001	2.136	3.029
Item 5	1 vs 0	0.780	0.065	12.00	<0.001	0.652	0.907
	2 vs 1	2.472	0.137	18.10	<0.001	2.204	2.740
Item 6	1 vs 0	0.725	0.064	11.24	<0.001	0.598	0.851
	2 vs 1	2.338	0.128	18.22	<0.001	2.087	2.590
Item 7	1 vs 0	0.387	0.061	6.35	<0.001	0.268	0.506
	2 vs 1	2.587	0.135	19.17	<0.001	2.323	2.852
Item 8	1 vs 0	1.849	0.090	20.57	<0.001	1.673	2.025
	2 vs 1	2.187	0.157	13.96	<0.001	1.880	2.494
Item 9	1 vs 0	0.597	0.063	9.49	<0.001	0.474	0.720
	2 vs 1	2.389	0.128	18.62	<0.001	2.138	2.641
Item 10	1 vs 0	0.928	0.068	13.60	<0.001	0.794	1.062
	2 vs 1	1.914	0.114	16.85	<0.001	1.691	2.137
Item 11	1 vs 0	1.792	0.088	20.35	<0.001	1.619	1.964
	2 vs 1	2.202	0.155	14.21	<0.001	1.898	2.505
Item 12	1 vs 0	0.172	0.061	2.81	0.005	.0519	0.292
	2 vs 1	1.840	0.099	18.61	<0.001	1.646	2.033
Item 13	1 vs 0	0.203	0.061	3.32	0.001	0.083	0.323
	2 vs 1	1.942	0.103	18.88	<0.001	1.741	2.144
Item 14	1 vs 0	−0.485	0.067	−7.60	<0.001	−0.610	−0.360
	2 vs 1	1.719	0.090	19.23	<0.001	1.544	1.894
Item 15	1 vs 0	−0.843	0.062	−1.35	0.177	−0.207	0.038
	2 vs 1	1.403	0.084	16.79	<0.001	1.239	1.567

Partial credit model, number of observations = 1329

Log likelihood = −14252.727

AIC = 28567.45

BIC = 28728.41

In three further IRT analyses of the partial credit model, all of the items within the three factors showed good fitness in the models. Although item 4 remained problematic, the three-factor solution was acceptable (see Table 6).

**Table 6:** Thresholds for the three possible factors of the PHQ-15 based on IRT analysis

	Diff.	Coef.	Std. Err.	z	*P* > z	[95 % Conf.	Interval]
Factor 1							
Item 2	1 vs 0	0.351	0.064	5.49	<0.001	0.226	0.477
	2 vs 1	2.094	0.114	18.31	<0.001	1.870	2.318
Item 3	1 vs 0	0.320	0.065	4.92	<0.001	0.193	0.448
	2 vs 1	1.577	0.095	16.52	<0.001	1.390	1.764
Item 4	1 vs 0	2.590	0.126	20.50	<0.001	2.342	2.838
	2 vs 1	2.685	0.237	11.32	<0.001	2.220	3.150
Item 5	1 vs 0	0.792	0.068	11.58	<0.001	0.658	0.926
	2 vs 1	2.567	0.145	17.71	<0.001	2.282	2.851
Item 6	1 vs 0	0.734	0.068	10.85	<0.001	0.602	0.867
	2 vs 1	2.427	0.136	17.83	<0.001	2.160	2.693
Item 7	1 vs 0	0.385	0.064	6.09	<0.001	0.261	0.509
	2 vs 1	2.684	0.144	18.61	<0.001	2.401	2.966
Item 8	1 vs 0	1.910	0.099	19.38	<0.001	1.716	2.103
	2 vs 1	2.277	0.164	13.90	<0.001	1.956	2.598
Item 11	1 vs 0	1.849	0.096	19.19	<0.001	1.661	2.038
	2 vs 1	2.292	0.162	14.14	<0.001	1.975	2.610
Factor 2							
Item 9	1 vs 0	0.366	0.040	9.16	<0.001	0.288	0.445
	2 vs 1	1.872	0.080	23.43	<0.001	1.715	2.029
Item 10	1 vs 0	0.558	0.042	13.18	<0.001	0.475	0.641
	2 vs 1	1.641	0.070	23.30	<0.001	1.503	1.779
Factor 3							
Item 1	1 vs 0	0.867	0.063	13.80	<0.001	0.745	0.992
	2 vs 1	2.052	0.112	18.37	<0.001	1.833	2.271
Item 12	1 vs 0	0.122	0.055	2.21	0.027	0.014	0.230
	2 vs 1	1.737	0.089	19.42	<0.001	1.561	1.912
Item 13	1 vs 0	0.152	0.055	2.76	0.006	0.044	0.260
	2 vs 1	1.831	0.093	19.67	<0.001	1.649	2.014
Item 14	1 vs 0	−0.469	0.057	−8.18	<0.001	−0.581	−0.357
	2 vs 1	1.598	0.081	19.64	<0.001	1.439	1.758
Item 15	1 vs 0	−0.118	0.056	−2.10	0.035	−0.228	0.008
	2 vs 1	1.329	0.075	17.62	<0.001	1.181	1.477

Partial credit model, number of observations = 1329

Factor 1: Log likelihood = −7025.6965; AIC = 14085.390; BIC = 14173.660

Factor 2: Log likelihood = −1942.3235; AIC = 3894.647; BIC = 3920.608

Factor 3: Log likelihood = −5613.5482; AIC = 11249.100; BIC = 11306.210

## Discussion

The present study evaluated the Chinese version of the PHQ-15 in a large tertiary hospital inpatient setting in Chengdu. The results revealed satisfactory reliability (Cronbach’s alpha = 0.83) of this scale and good evidence of its validity. Cronbach’s alpha in this study was higher than that in Western and Chinese studies (between 0.78 and 0.82).

The correlations of the PHQ-15 scores with the PHQ-9 depression scale and the GAD-7 anxiety scale scores were similar to the correlations between these instruments in other studies; this evidence suggests that the PHQ-15 has discriminant validity [31].

The correlations of the PHQ-15 score with the PHQ-9 and GAD-7 scale scores were not sufficiently high to completely attribute the PHQ-15 results to coexisting depressive and anxiety symptoms. Aside from medical comorbidities, functional or bodily distress symptoms were observed as factors (discriminant validity).

In a factor analysis of a former version of the PHQ-15 in a USA clinical study, three factors were identified: cardiopulmonary, gastrointestinal, and general pain/fatigue (explanation of the total variance: 46 %) [32]. A study from Hong Kong [18] determined four clinically meaningful factors that explained 49.7 % of the total variance: “cardiopulmonary,” “gastrointestinal,” “pain” and “neurological”. A study from Shanghai [19] identified three factors: “general discomfort,” “gastro-intestinal discomfort” and “cardiothoracic discomfort” (explanation of the total variance: 54 %). Based on factorial analysis in our study, we identified three factors, referred to as “cardiopulmonary” “gastrointestinal” and “pain/neurological,” which explained 56 % of the total variance. A second-order factor analysis including these three factors produced an acceptable model. Because of substantial double-factor loadings, a unidimensional model is also discussed.

Item 4 (menstrual problems), item 8 (sexual problems) and item 11 (fainting spells) displayed extreme floor effects. These floor effects were also found in previous Chinese and Western studies [18, 19, 21, 29, 33, 34]. Additionally, item 4 displayed a very small variance of 0.35 and showed very small differences in its thresholds based on IRT analysis.

Because of their limited associations with other items, rare symptom prevalence, and limited associations with measures of functioning, quality of life, and health service use, these three items were not included in a new questionnaire, termed “The Somatic Symptom Scale-8” (SSS-8) [35].

### Strengths and limitations

This is the first validation study of the PHQ-15 in a large sample of patients at a major Chinese tertiary hospital that has a full complement of services for a broad range of medical conditions.

The sample included the most important departments of a general hospital. The patients were representative of general hospital inpatients with respect to sex, marital status, education level and income level. The overall response rate was very high (90.1 %). The validation process included IRT analysis, which is a new analysis of the PHQ-15.

However, there were some limitations of our study. (1) We did not perform a structured clinical interview; therefore, the sensitivity and specificity of the PHQ-15 for assessing somatoform disorders could not be established. However, the PHQ-15 is best characterized as a measure of somatic symptom severity rather than a diagnostic instrument for somatoform disorders [5]. It would be important to diagnose patients with a new classification system of somatic symptom disorders in China. (2) The study was cross-sectional. Longitudinal studies are needed to determine the test-retest reliability of the PHQ-15 and its responsiveness to treatment. (3) There was no assessment of functional status or health-related quality of life. (4) There was no systematic assessment of medical conditions or independent measure of healthcare utilization. (5) Indigenous and common expressions of somatic distress among Chinese patients are not captured by the PHQ-15. (6) A multi-center study would be an optimal approach.

## Conclusions

The PHQ-15 displayed adequate reliability and good evidence of validity for detecting patients with severe somatic symptoms in a Chinese hospital. Several of the current findings were consistent with previous research regarding the PHQ-15. To improve the diagnostic quality of the PHQ-15, items 4, 8 and 11 can be omitted.

Future research should examine whether differences in factorial structure and the cross-loading of items across populations are related to sampling, methodological factors and/or cultural differences in experiences with somatic disorders.

## Ethics approval and consent to participate

The study was approved by the Ethics Committee of West China Hospital of Sichuan University. Written informed consent was obtained from all participants.

## Consent for publication

Not applicable.

## Availability of data and materials

All the data supporting our findings is contained within the manuscript.

## Additional file

Additional file 1: PHQ-15 Chinese version. (DOCX 15 kb)

## Abbreviations

AIC: Akaike information criterion
BIC: Bayesian information criterion
CFI: comparative fit index
DSM-5: diagnostic and statistical manual of mental disorders 5th edition
GAD-7: general anxiety disorder -7
IBM SPSS: statistics
IRT: item response theory
MANOVA: multivariate analysis of variance
PHQ-15: patient health questionnaire -15
PHQ-9: patient health questionnaire -9
PRIME-MD: primary care evaluation of mental disorders
PRIME-MD PHQ: primary care evaluation of mental disorders- patient health questionnaire
RMSEA: root mean square error of approximation
SD: standard deviation
SOM: somatoform symptom
SRMR: standardized root mean square residual
SSD: somatic symptom disorders
SSS: somatic symptom severity
TLI: Tucker-Lewis index
SPSS: MPlus and Stata are statistic programms
