# Supplementary material for: Validation of the Chinese version of the PHQ-15 in a tertiary hospital
Source: BMC Psychiatry. 2016 Apr 5;16:89. doi: 10.1186/s12888-016-0798-5 (PMC4820992; doi:10.1186/s12888-016-0798-5)
Supplement: Additional file 1: — PHQ-15 Chinese version. (DOCX 15 kb) [file 12888_2016_798_MOESM1_ESM.docx]

**躯体症状(PHQ-15)**

**在过去4星期，您受到以下任何问题所困扰的程度有多少？**

|  | **没有困扰** | **少许困扰** | **很多困扰** |
| --- | --- | --- | --- |
| a. 胃痛或肚痛 | 0 | 1 | 2 |
| b. 背痛 | 0 | 1 | 2 |
| c. 手臂、腿、或关节（膝盖、髋部等）的疼痛 | 0 | 1 | 2 |
| d. 月经痛或其它与月经有关的问题（仅供女士作答） | 0 | 1 | 2 |
| e. 头痛 | 0 | 1 | 2 |
| f. 胸痛 | 0 | 1 | 2 |
| g. 晕眩 | 0 | 1 | 2 |
| h. 短时间晕倒 | 0 | 1 | 2 |
| i. 感到心脏砰砰跳动或跳得很快 | 0 | 1 | 2 |
| j. 透不过气来 | 0 | 1 | 2 |
| k. 性交时的疼痛或问题 | 0 | 1 | 2 |
| l. 便秘，稀便或腹泻 | 0 | 1 | 2 |
| m. 恶心，胀气或消化不良 | 0 | 1 | 2 |
| n. 感觉疲劳或无精打采 | 0 | 1 | 2 |
| o. 睡眠问题或烦恼 | 0 | 1 | 2 |
